# COVID-19 in 16 West African Countries: An Assessment of the Epidemiology and Genetic Diversity of SARS-CoV-2 after Four Epidemic Waves

**DOI:** 10.4269/ajtmh.22-0469

**Published:** 2023-08-28

**Authors:** Anna Julienne Selbé Ndiaye, Mamadou Beye, Aissatou Sow, Gora Lo, Abdou Padane, Cheikh Sokhna, Coumba Touré Kane, Philippe Colson, Florence Fenollar, Souleymane Mboup, Pierre-Edouard Fournier

**Affiliations:** ^1^ Institut de Recherche en Santé, de Surveillance Epidémiologique et de Formation, Dakar, Senegal;; ^2^ Institut Hospitalo-Universitaire-Méditerranée Infection, Marseille, France;; ^3^ Vecteurs - Infections Tropicales et Méditerranéennes, Campus International Institut de Recherche pour le Développement-Université Cheikh Anta Diop de l’IRD, Dakar, Senegal;; ^4^ IRD, Assistance Publique - Hôpitaux de Marseille, Service de Santé des Armées, VITROME, Aix Marseille University, Marseille, France;; ^5^ IRD, AP-HM, Microbes Evolution Phylogeny and Infections, Aix Marseille University, Marseille, France

## Abstract

West Africa faced the COVID-19 pandemic in early March 2020 and, as of March 31, 2022, had more than 900,000 confirmed cases and more than 12,000 deaths. During this period, SARS-CoV-2 genomes evolved genetically, resulting in the emergence of distinct lineages. This review was conducted to provide the epidemiological profile of COVID-19, the mutational profile of SARS-CoV-2, and the dynamics of its lineages in the 16 west African countries by analyzing data from 33 studies and seven situation reports. For a more complete representation of the epidemiology and genetic diversity of SARS-CoV-2, we used reliable public data in addition to eligible studies. As of March 31, 2022, the 16 west African countries experienced four epidemic waves with variable intensities. Higher mortality was noted during the third wave with a case fatality rate (CFR) of 1.9%. After these four epidemic waves, Liberia recorded the highest CFR (4.0%), whereas Benin had the lowest CFR (0.6%). Through mutational analysis, a high genetic heterogeneity of the genomes was observed, with a predominance of mutations in the spike protein. From this high mutational rate, different lineages emerged. Our analysis of the evolutionary diversity allowed us to count 205 lineages circulating in west Africa. This study has provided a good representation of the mutational profile and the prevalence of SARS CoV-2 lineages beyond the knowledge of the global epidemiology of the 16 African countries.

## INTRODUCTION

In December 2019, a pneumonia cluster of unknown etiology appeared in Wuhan City, China.^1^ The WHO was notified, and then 2019-nCov, a novel coronavirus, was characterized as the causative agent of this pneumonia, which subsequently became an epidemic in Wuhan.^2^^,^^3^ As of June 28, 2023, according to data reported by the WHO, the world had recorded more than 768,187,096 confirmed cases and more than 6,945,714 deaths related to COVID-19.^4^

In Africa, the first COVID-19 case was detected in Egypt on February 14, 2020 and had reached the continent via travelers returning from hot spots in Asia, Europe, and the United States (https://africacdc.org/).^5^ To date, all African countries have been affected by the pandemic. As of June 28, 2023, the WHO African region had recorded more than 9,538,679 confirmed cases and more than 175,394 deaths.^6^ However, if COVID-19 initially progressed less rapidly in Africa than in other parts of the world, it really accelerated on this continent with the circulation of variants of concern (VOCs). Thus, most African countries have seen their case-fatality rate (CFR) exceed the world average of 2.2%.^7^ With the emergence of these variants, continuous sequencing of the virus has become necessary to monitor new variants and provide key elements in the response to the COVID-19 pandemic. In view of the upheaval caused by this disease and the unpredictable viral evolution of SARS-CoV-2, this review aims to synthesize the different aspects of the COVID-19 pandemic in the west African region to identify future surveillance needs in this part of Africa. This will be done through a synthesis of current knowledge on the epidemiology of COVID-19, mutational analysis, and genetic evolution of SARS-CoV-2 in west Africa.

## MATERIALS AND METHODS

We conducted a literature search in three electronic databases (PubMed, Google Scholar, and Web of Sciences), focusing on the epidemiology and genetic evolution of SARS-CoV-2 in west Africa. We used the following research themes: “COVID-19 in west Africa,” “coronavirus disease and Africa,” “epidemiology, SARS-CoV-2, Africa,” “coronavirus and Africa,” “variants of SARS-CoV-2 and west Africa,” and “lineages, SARS-CoV-2, west Africa.” For the research articles, only studies available in full text free of charge or as a review in English or French were selected. To analyze the epidemiological data of the four phases of the pandemic in 16 west African countries, we used the COVID19R package, available in the Comprehensive R Archive Network and hosted on GitHub6.^8^ This package aggregates several data from reliable sources, including the Johns Hopkins University Center for Systems Science and Engineering. The data are harmonized and put in comma separated values (CSV) format. For the epidemiological data, we looked at confirmed cases and deaths recorded from February 28, 2020 to March 31, 2022 in the 16 west African countries. In addition to this package and literature search, we also searched WHO data through its situation report and real-time data site. Available disease status reports for selected countries were also used.

For the study of genetic diversity in west Africa, in addition to reviewing studies that have focused on the genetics of SARS-CoV-2, we analyzed sequence data from 8,650 genomes deposited on GISAID by the 16 west African countries. These genomes can be accessed under the GISAID identifier EPI_SET_230426sc https://doi.org/10.55876/gis8.230426sc. We uploaded complete, high-coverage sequences defined as sequences with less than 1% unidentified nucleotides and less than 0.05% unidentified mutations in another isolate and no unverified indel mutations by the submitter. Using the Pangolin nomenclature, which combines genetic and geographic components, we established chronologically the different lineages of SARS-CoV-2 that circulated in west Africa between February 28, 2020 and March 31, 2022.

## RESULTS

### Description of included studies.

The database search included 33 studies and seven situation reports conducted in west Africa (Table 1). The situation reports provided updates on the epidemiological profile (confirmed cases and deaths) of the COVID-19 pandemic across different west African countries. Of the 33 research articles included, 10 addressed the epidemiology of COVID-19 and five were seroprevalence studies. Eighteen studies addressed genomic and mutational analysis and lineage dynamics of SARS-CoV-2 in west Africa.

**Table 1:** Characteristics of the studies and status reports included

*N*	Study ID [Ref.]	Country of study	Period of study	Hyperlinks
1	NCDC^9^	Nigeria	February 2020	https://ncdc.gov.ng/themes/common/files/sitreps/34a2340028bf9a6079f1ec7ff431612b.pdf
2	Organization for Economic Co-operation and Development^10^	West African countries	February 2020–March 2022	coronavirus-ouest-afrique - Club du Sahel et de l’Afrique de l’Ouest (CSAO) (oecd.org)
3	Dalal et al.^11^	Sub-Saharan African countries	February–September 2020	https://www.ncbi.nlm.nih.gov/pmc/articles/PMC8611236/
4	Skrip et al.^12^	Sub-Saharan African countries	May 2020	https://www.ncbi.nlm.nih.gov/pmc/articles/PMC7518969/
5	IMMAP^13^	Burkina Faso	March 2020–September 2021	https://immap.org/product/covid-19-situational-analysis-annual-report-for-burkina-faso-context-march-2020-september-2021/
6	World Health Organization^14^	Ivory Coast	January–November 2020	https://www.afro.who.int/sites/default/files/2021-04/Rapport%20de%20documentation%20de%20la%20riposte_BON%283%29_0.pdf
7	Kenu et al.^15^	Ghana	March–June 2020	https://www.ncbi.nlm.nih.gov/pmc/articles/PMC8087358/
8	Donamou et al.^16^	Guinea Conakry	March–July 2020	https://www.ncbi.nlm.nih.gov/pmc/articles/PMC7859622/
9	Oumar et al.^17^	Mali	April–October 2020	https://search.bvsalud.org/aimafro/resource/en/biblio-1283943
10	Ahmed et al.^18^	Mauritania	December 2020	https://jidc.org/index.php/journal/article/view/34516409
11	Elimian et al.^19^	Nigeria	February–June 2020	https://pubmed.ncbi.nlm.nih.gov/33334842/
12	Akande et al^20^	Nigeria	February–April 2021	https://gh.bmj.com/content/6/11/e007076
13	Majiya et al.^21^	Niger	June 2020	https://www.medrxiv.org/content/10.1101/2020.08.04.20168112v2
14	Olayanju et al.^22^	Nigeria	December–April 2020	https://www.ajtmh.org/view/journals/tpmd/104/1/article-p91.xml
15	Ifeorah et al.^23^	Nigeria	August 2020	https://www.researchsquare.com/article/rs-151037/v1
16	Milleliri et al.^24^	Ivory Coast	July–October 2020	https://www.ncbi.nlm.nih.gov/pmc/articles/PMC8103493/
17	Barrie et al.^25^	Sierra Leone	March 2021	https://gh.bmj.com/content/6/11/e007271.long
18	Garenne^26^	African countries	February–August 2020	ferdi-wp-271-l-ironie-du-corona-epidemie-de-covid-19-et-developpement-en.pdf (hal.science)
19	World Health Organization^27^	Multiple countries	October 2020	https://www.who.int/publications/m/item/weekly-epidemiological-update—27-october-2020
20	LO et al.^28^	Multiple countries	September 2020	https://www.africaportal.org/publications/la-covid-19-en-afrique-bilan-detape-et-perspectives-covid-19-in-africa-progress-report-and-prospects/
21	Kouriba et al.^29^	Mali	April 2020	https://www.mdpi.com/1999-4915/12/11/1251
22	Sylverken et al.^30^	Ghana	February–March 2020	https://link.springer.com/article/10.1007/s00705-021-04986-3
23	Ahouidi et al.^31^	Senegal	March 2020–March 2021	https://www.nature.com/articles/s41598-021-02874-z
24	Grayo et al.^32^	Guinea Conakry	March 2020–July 2021	https://www.ncbi.nlm.nih.gov/pmc/articles/PMC8798712/
25	Sander et al.^33^	Benin	January–April 2021	https://www.ncbi.nlm.nih.gov/pmc/articles/PMC8544961/
26	Ozer et al.^34^	Nigeria	July 2020–August 2021	https://www.nature.com/articles/s41467-022-28317-5
27	Lin et al.^35^	Sierra Leone	December 2020–August 2021	https://www.sciencedirect.com/science/article/pii/S1567134822000053
28	World Health Organization^36^	West African countries	August 2021	https://www.afro.who.int/news/west-africa-covid-19-deaths-surge-amid-ebola-and-other-outbreaks
29	Yadouleton et al.^37^	Benin	April–July 2021	https://wwwnc.cdc.gov/eid/article/28/1/21-1909_article
30	Happi et al.^38^	Nigeria	February 2020	https://virological.org/t/first-african-sars-cov-2-genome-sequence-from-nigerian-covid-19-case/421
31	Happi et al.^39^	Nigeria	May 2020	https://virological.org/t/sars-cov-2-genomes-from-nigeria-reveal-community-transmission-multiple-viruslineages-and-spike-protein-mutation-associated-with-higher-transmission-and-pathogenicity/494
32	Taiwo et al.^40^	Nigeria	July 2020	https://www.sciencedirect.com/science/article/pii/S2052297522000075
33	Ngoi et al.^41^	Ghana	March–May 2020	https://www.ncbi.nlm.nih.gov/pmc/articles/PMC7746953/
34	Morang’a et al.^42^	Ghana	June–September 2021	https://www.researchsquare.com/article/rs-1088719/v1
35	Kritsky et al.^43^	Guinea Conakry	May, June 2020–March 2021	https://www.biorxiv.org/content/10.1101/2021.07.28.454098v1
36	Dara et al.^44^	Mali	June 2020	https://www.mdpi.com/2227-9717/9/12/2169
37	Pirnay et al.^45^	Niger	May 2020	https://www.ncbi.nlm.nih.gov/pmc/articles/PMC7552053/
38	Sanyang et al.^46^	Gambia	March 2021	https://www.thelancet.com/journals/langlo/article/PIIS2214-109X(21)00213-8/fulltext
39	World Health Organization^47^	African countries	March 2022	https://au.int/sites/default/files/documents/41648-doc-AfricaCDC_COVIDBrief_29March22_FR.pdf
40	Wruck and Adjaye^48^	West African countries	December 2019–April 2020	https://www.nature.com/articles/s41598-021-00267-w

### COVID-19 in west Africa.

**Figure 1.**
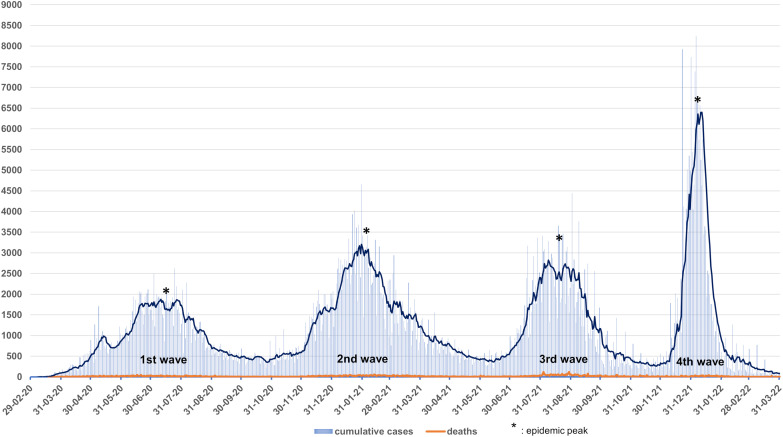
Evolution of confirmed cases and deaths in west Africa as of March 31, 2022.

**Figure 2.**
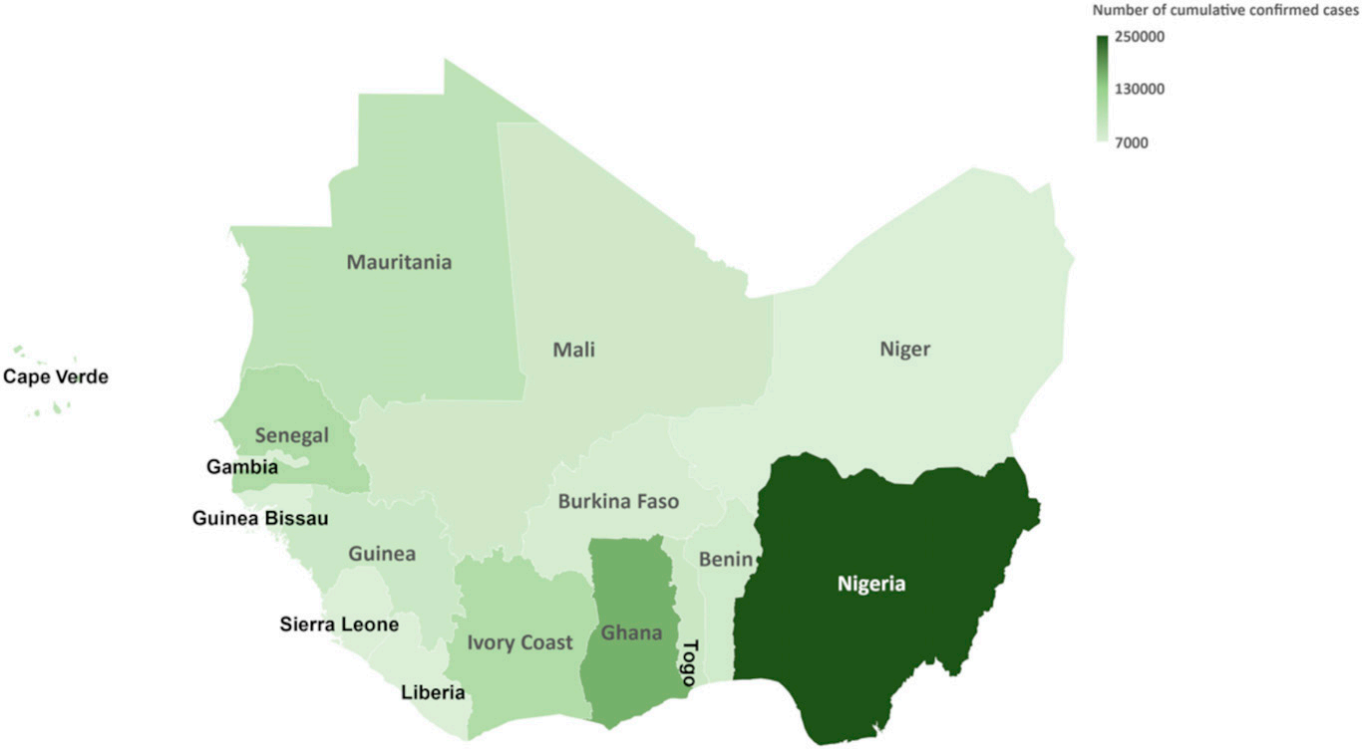
Map of west Africa with the number of cases as of March 31, 2022.

The west African region faced four epidemic waves between February 2020 and March 2022. The first epidemic wave started in the western part of the African continent with the first case recorded in Lagos State, Nigeria, on February 27, 2020. The patient was an Italian citizen coming from Milan.^9^ After this index case, the evolution of the pandemic in west Africa was rapid, with the presence of the virus in all 16 countries of the region within a month.^10^ The peak of the first phase occurred in the third week of July 2020. The first epidemic wave of COVID-19 spread across west Africa between February and October 2020. The second wave was from November 2020 to May 2021. The detection of new cases of the disease during this second wave peaked between January and February 2021. The third wave began in June 2021, with an epidemic peak reached in August 2021. The period of this third wave in west Africa was considered between June and November 2021. In the first week of December 2021, an upward trend in new cases was reported, reflecting the start of the fourth epidemic wave. This fourth wave reached its epidemic peak in the first week of January 2022. From December 2021 to March 2022, west Africa experienced a fourth COVID-19 epidemic wave. As of March 31, 2022, after four epidemic waves (Figure 1), approximately 894,341 confirmed COVID-19 cases and more than 11,900 deaths had been reported in the 16 west African countries (Figure 2, Table 2).

**Table 2:** Cumulative cases and deaths in the 16 west African countries as of March 31, 2022

Country	Cumulative confirmed cases (*n*)	Cumulative deaths (*n*)	CFR (%)	Confirmed cases per 100,000 population
Benin	26,952	163	0.60	201
Burkina Faso	20,853	381	1.83	92
Cape Verde	55,952	402	0.72	9,483
Ivory Coast	81,741	795	0.97	290
Gambia	11,988	365	3.04	444
Ghana	160,971	1,445	0.90	481
Guinea	36,459	438	1.20	262
Guinea-Bissau	8,149	170	2.09	388
Liberia	7,400	294	3.97	140
Mali	30,484	727	2.38	135
Mauritania	58,669	982	1.67	1,248
Niger	8,801	308	3.50	34
Nigeria	255,414	3,140	1.23	117
Senegal	85,895	1,962	2.28	497
Sierra Leone	7,674	125	1.63	89
Togo	36,939	272	0.74	420
Total	894,341	11,969	–	–

CFR = case fatality rate.

Despite few studies oriented on the impact of SARS-CoV-2 infection according to gender and age groups, some epidemiological studies demonstrated that women have been less exposed to SARS-CoV-2 infection than men and exhibited lower mortality rates. One hundred and twenty days after the onset of the disease in sub-Saharan Africa, a study conducted in 20 countries including eight west African countries showed that women accounted for 38.1% of total reported cases compared with 61.9% for men and for 29.4% of total deaths compared with 70.5% among men. Deaths were more frequently reported in those 60 years and older, with 51.3% of deaths in this age group.^11^ This same trend was found in a preliminary analysis of epidemiological data from 28 African countries conducted by the WHO, which showed that women accounted for about 41% of COVID-19 cases. In west Africa, after the actual spread of the pandemic, this gender-specific difference in SARS-CoV-2 infection and mortality in subjects over 60 years of age was confirmed and demonstrated by epidemiological data from many west African countries, including Burkina Faso,^12^^,^^13^ Ivory Coast,^14^ Ghana,^15^ Guinea,^16^ Mali,^17^ Mauritania,^18^ and Nigeria.^19^^,^^20^ Studies exploring population exposure have been conducted in some west African countries. In Nigeria, the seroprevalence was evaluated within specific populations in asymptomatic subjects and made it possible to estimate seroprevalence rates between 2.2% and 45%.^21^^–^^23^ In Ivory Coast, a seroprevalence of 14–35% was found in an asymptomatic population of artisanal gold miners.^24^ A cross-sectional serological survey conducted in Sierra Leone found an overall weighted seroprevalence of 2.6%, representing a prevalence 43 times higher than the cases reported in the country.^25^ A meta-analysis carried out in 2021 estimated a seroprevalence of 25% in west Africa.^49^

### Epidemiological profile of COVID-19 and genetic diversity of SARS-CoV-2 in the first wave in west Africa.

For this first wave, the highest number of new cases in west Africa was recorded on July 24, 2020. To date, more than 123,000 cumulative cases have been reported in west Africa, with Nigeria representing the most affected country (39,539 confirmed cases; 31.9% of all cases in the region). As of August 15, 2020, west Africa was among the least affected African regions, with a cumulative incidence of 362 cases per million population and a case-fatality of 16 per 1,000 cases, behind southern Africa, north Africa, and the African islands.^26^ The downward phase of the first epidemic wave was marked by a 36% decrease in new cases in August 2020 compared with the epidemic peak in July 2020. This downward trend continued until October 2020, marking the end of the first wave of COVID-19 in west Africa. Approximately 2,930 deaths were recorded during this first phase of the disease, resulting in a CFR of 1.4%,^27^ a rate that, like that of the continent, was relatively low during this first wave compared with other regions of the world. During this first epidemic wave in west Africa, a relatively high cure rate of 90% was observed.^28^ The highest mortality rates were recorded in Liberia and Niger, with 5.8% and 5.7%, respectively (Table 3).

**Table 3:** Distribution of new cases, new deaths, and case-fatality rates in the 16 west African countries as of March 31, 2022

Country	1st wave			2nd wave			3rd wave			4th wave		
	New cases (*n*)	New deaths (*n*)	CFR (%)	New cases (*n*)	New deaths (*n*)	CFR (%)	New cases (*n*)	New deaths (*n*)	CFR (%)	New cases (*n*)	New deaths (*n*)	CFR (%)
Benin	2,643	41	1.55	5,415	60	1.11	16,792	60	0.36	2,102	2	0.10
Burkina Faso	2,500	67	2.68	10,934	99	0.91	2,566	119	4.64	4,853	96	1.98
Cape Verde	8,793	95	1.08	21,646	169	0.78	7,931	86	1.08	17,582	52	0.30
Ivory Coast	20,716	126	0.61	26,576	179	0.67	14,432	398	2.76	20,017	92	0.46
Gambia	3,672	119	3.24	2,321	60	2.59	3,996	163	4.08	1,999	23	1.15
Ghana	48,055	320	0.67	45,843	465	1.01	37,022	424	1.15	30,051	236	0.79
Guinea	12,072	72	0.60	11,105	88	0.79	7,593	225	2.96	5,689	53	0.93
Guinea-Bissau	2,413	41	1.70	1,353	27	2.00	2,674	80	2.99	1,709	22	1.29
Liberia	1,426	82	5.75	765	4	0.52	3,633	201	5.53	1,576	7	0.44
Mali	3,554	136	3.83	10,713	381	3.56	3,167	89	2.81	13,050	121	0.93
Mauritania	7,703	163	2.12	11,844	300	2.53	19,719	369	1.87	19,403	150	0.77
Niger	1,220	69	5.66	4,195	123	2.93	1,592	67	4.21	1,794	49	2.73
Nigeria	62,852	1,144	1.82	103,665	953	0.92	47,700	878	1.84	41,197	165	0.40
Senegal	15,616	324	2.07	25,800	814	3.16	32,571	745	2.29	11,908	79	0.66
Sierra Leone	2,366	74	3.13	1,781	5	0.28	2,255	42	1.86	1,272	4	0.31
Togo	2,331	57	2.45	11,135	68	0.61	12,799	118	0.92	10,674	29	0.27

CFR = case fatality rate.

**Figure 3.**
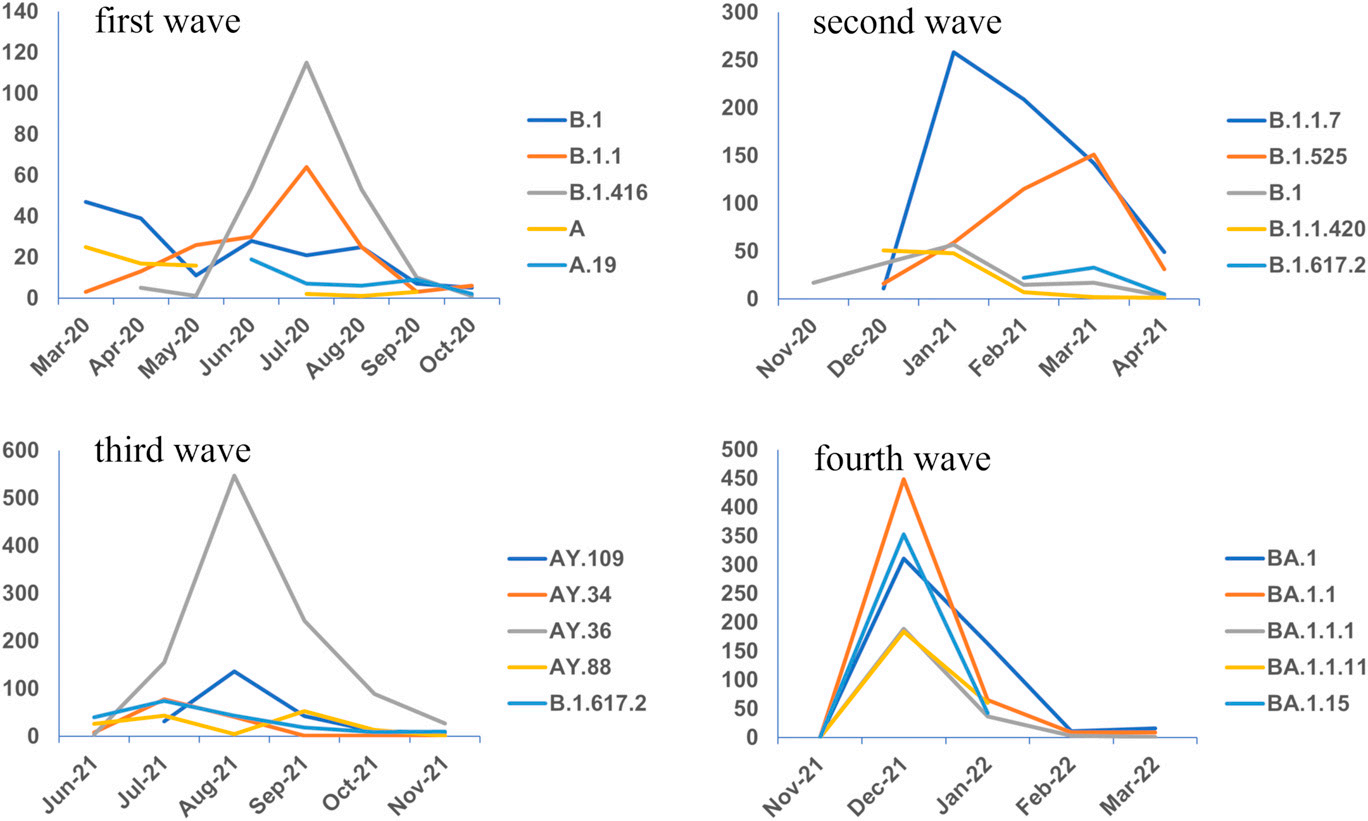
Chronological distribution of the majority lineages over the different waves.

During the first phase of COVID-19 in west Africa, the first sequences analyzed showed the circulation of two distinct viral lineages: A and B.^29^^,^^30^^,^^41^ These same lineages were found through the analysis of 1,016 complete and high-coverage genomes that were deposited by west African countries (Supplemental Table 5), except Cape Verde, Liberia, and Mauritania, in public database GISAID (epicov.org/epi3/frontend). Senegal, the Gambia, and Nigeria were the countries that produced the highest number of complete and high coverage sequences during this first wave with 26.3% (*N* = 267), 22.9% (*N* = 233), and 18.9% (*N* = 192) of the sequences, respectively. From the genomic data in this first wave, 64 circulating SARS-CoV-2 lineages in west Africa were observed (Supplemental Table 6), 15 of which represented 1–23% of sequences (Supplementary Figure 1). Lineage B.1.416 (23.5% of all sequences) was the dominant lineage, followed by lineages B.1 (18.0%), B.1.1 (16.7%), A (6.3%), and A.19 (4.2%). Lineage B.1, predominant in March and April 2020 (Figure 3), circulated in all west African countries that studied SARS-CoV-2 genetic diversity except Guinea Bissau (Supplemental Table 1). In May 2020, lineage B.1.1 that had emerged in March 2020 became the most common lineage (Figure 3) except in Benin, Côte d’Ivoire, and Mali (Supplemental Table 1). The B.1.416 lineage, characterized as the Senegalese lineage (https://cov-lineages.org/lineage_list.html), was the majority lineage of the first wave. This may be biased by the fact that most of the whole-genome sequences in the first phase of the epidemic originated from Senegal. In Senegal, lineage B.1.416 represented 59.4% of sequenced strains.^31^ No lineage corresponding to a SARS-CoV-2 VOC circulated during this first epidemic wave in west Africa.

### Epidemiological profile of COVID-19 and genetic diversity of SARS-CoV-2 in the second wave in west Africa.

A resurgence of COVID-19 cases was noted at the beginning of November 2020, marking the beginning of a second epidemic wave. The epidemic peak was recorded between January and February, with an average of 2,544 daily cases of COVID-19. Burkina Faso, Nigeria, and Sierra Leone experienced their epidemic peak in January 2021, with 36.3%, 42.2%, and 55.1% of their cases, respectively. Benin, Ghana, Guinea Bissau, and Senegal experienced their epidemic peak in February 2021. These countries recorded 1.8%, 15.4%, 0. 5%, and 8.7% of total COVID-19 cases, respectively, recorded during this second wave.

However, some individual countries such as Mauritania and Niger reached their epidemic peak earlier, in December 2020, or later, in March 2021 for Ivory Coast, the Gambia, Guinea, and Togo and in April 2021 for Cape Verde. Mauritania recorded a total of 11,844 cases, with 48.9% of cases recorded in December and a daily average of 185 new cases. Niger recorded 4,195 cases during its second epidemic wave, with a CFR of 2.9%. In Liberia, the second epidemic wave was less pronounced than in other west African countries. Indeed, between November 2020 and May 2021, the country recorded an average of four cases per day, with 765 confirmed cases and four deaths and a CFR of 0.5%.

A particularity during this second wave in the west of the continent was Mali; between November 2020 and May 2021, Mali faced two fulgurating waves of new contaminations: in December 2020 and April 2021 with a lull in February 2021. The country experienced a record increase of 300% of confirmed cases compared with the first wave.

During the month of May 2021, the west African region experienced a downward trend in new cases of the disease. As of May 31, 2021, west Africa reported 295,091 new confirmed cases and 3,795 new deaths. The CFR was estimated at 1.3%.

Nigeria (103,665 cases; 34.7% of all cases in west Africa), Ghana (45,843 cases; 15.4%), Ivory Coast (26,576 cases; 8.9%), Senegal (25,800 cases; 8.6%), and Cape Verde (21,646 cases; 7.3%) were the five countries with the largest numbers of new cases in west Africa.

The following countries recorded less than 5% of second wave cases: Liberia (765 cases; 0.3%), Guinea Bissau (1,353 cases; 0.5%), Sierra Leone (1,781 cases; 0.6%), the Gambia (2,321; 0. 8%), Niger (4,195 cases; 1.4%), Benin (5,415 cases; 1.8%), Mali (10,713 cases; 3.6%), Burkina Faso (10,934 cases; 3.7%), Guinea (11,105 cases; 3.7%), Togo (11,135 cases; 3.7%), and Mauritania (11,844 cases; 4.0%).

Nigeria (953 deaths; 24.6% of all deaths), Senegal (814 deaths; 21.0%), Ghana (465 deaths; 12.0%), Mali (381 deaths; 9.8%), and Mauritania (300 deaths; 7.8%) were the countries with the highest mortality during the second wave. When the death numbers were compared with those of the first wave, record increases were reported in countries such as Mali (180% increase), Mauritania (84% increase), Niger (78% increase), and Senegal (150%).

Of the 16 west African countries, the following six countries had higher CFRs than the whole west African region (1.3%): Mali (3. 6%), Senegal (3.2%), Niger (2.9%), the Gambia (2.6%), Mauritania (2.5%), and Guinea-Bissau (2.0%) (Table 3).

Compared with the first wave, this second epidemic wave saw a more important evolution of confirmed cases (+49%) as well as deaths (+29%).

With the genetic evolution of SARS-CoV-2 during its expansion and social exchanges between countries causing the virus to migrate, several variants emerged. In west Africa, during the second epidemic wave, VOCs and variants of interest (VOIs) predominated. In several west African countries, the VOC Alpha and the VOI Eta constituted the two main competing variants.^31^^–^^34^ These data were confirmed by the analysis of west African sequences deposited in GISAID. From November 2020 to May 2021, 1,901 complete and high-coverage genomes were deposited by the 16 west African countries (Supplemental Table 5). This resulted in 67 SARS-CoV-2 lineages being identified, including 42 new lineages (Supplemental Table 6). Among these, VOC Alpha was the majority lineage (35% of all sequences), followed by VOI Eta (20%), B.1 (8%), B.1.1.420 (6%), and the VOC Delta (3%) (Supplemental Figure 1). The VOC Alpha emerged in December 2020 and was more prevalent in January and February 2021 (Figure 3), coinciding with the increased incidence of the epidemic in west Africa. The VOC Alpha was detected in all west African sequences except Mali and Sierra Leone (Supplemental Table 2). A study conducted in Sierra Leone effectively showed that the R.1 lineage was responsible for the second wave of infections in the country and that no VOC or VOI was detected.^35^ The second major lineage of this second wave, the VOI Eta, emerged in December 2020 and was more widespread in March 2021. In contrast, this lineage was not identified among sequences from Cape Verde, the Gambia, Guinea-Bissau, Mauritania, and Sierra Leone, which were the countries with the lowest numbers of whole-genome sequences reported. This may be a cause of underrepresentation of the genetic diversity of the virus in these countries. Delta was the second predominant VOC in the second wave. Discovered in October 2020 in India, the first west African sequences of VOC Delta were obtained in February 2021 (Figure 3). Early in its emergence, this VOC was detected in west Africa from sequences in Cote d’Ivoire, Ghana, Liberia, and Togo (Supplemental Table 2).

### Epidemiological profile of COVID-19 and genetic diversity of SARS-CoV-2 in the third wave in west Africa.

In the third week of June 2021, a recrudescence of confirmed cases was noted in west Africa, with 3,207 new cases, an increase of 23% compared with the second week. During this peak period, a significant increase in death numbers was reported in the region. Indeed, compared with the 4 weeks prior to the peak, the number of deaths increased by 193%, from 348 deaths to 1,018 deaths by mid-August 2021.^36^ The number of new cases decreased (−37%) during the month of September 2021 compared with August. This downward trend persisted until the last week of November 2021. During this wave, the number of new cases in the west African region decreased slightly (−26%) by comparison with the second wave, but a 1.9% CFR, higher than during the second wave (1.3%), was recorded. In detail, these trends varied from country to country.

Compared with the second wave, the Gambia, Guinea Bissau, Liberia, Sierra Leone, and Togo experienced a larger and more deadly third wave, with record increases in confirmed cases of 72%, 97%, 374%, 26%, and 14%, respectively, compared with the second wave and CFRs of 4.1%, 3.0%, 5.5%, 1.9%, and 0.9%, respectively. In contrast, compared with the second wave, a simultaneous decrease in new cases and deaths was noted in Mali and Niger. Despite a decrease in cases during the third wave, CFRs increased in Burkina Faso, Cape Verde, Ivory Coast, Ghana, Guinea and Nigeria. Mauritania and Senegal experienced case number increases of 66% and 26%, respectively; however, a slight decrease in case fatality occurred in these two countries. According to the data reported for Benin, there was no variation in reported deaths during the second and third waves. Despite new cases peaking with a 210% increase during the third wave in this country, a low CFR was recorded (Table 3).

The third epidemic wave was marked by an increase in sequencing capacity in west Africa. Sequence data extracted from complete genomes with high coverage showed increases of 130% and 23%, respectively, compared with sequences collected in the first and second waves. Between June 2021 and November 2021, 2,338 west African sequences (complete genomes) were produced (Supplemental Table 5), excluding sequences from Guinea Bissau and Burkina Faso. From the analysis of these sequences and according to the dynamics of the Pangolin nomenclature, 63 lineages circulated during this period (Supplemental Table 6), including 47 sub-lineages issued from the Delta VOC. This VOC has been associated with an increase in cases in several west African countries.^32^^,^^37^^,^^50^ Sub-lineage AY.36, also referred to as sub-lineage B.1.617.2.36, was the majority lineage in the third epidemic wave in west Africa (45% of all sequences), followed by sub-lineages AY.34 (10%), AY.88 (8%), B.1.617.2 (6%), and AY.109 (6%) (Supplemental Figure 1). Sub-lineage AY.36, was among the new lineages in this third wave and emerged in June 2021. It was the main sub-lineage between July and November 2021 (Figure 3), notably in Nigeria, that produced 59% of all complete and high-coverage genomes in the third wave. The various Delta-deriving AY sub-lineages were present across different west African countries (Supplemental Table 3).

### Epidemiological profile of COVID-19 and genetic diversity of SARS-CoV-2 in the fourth wave in west Africa.

The fourth epidemic wave started with an upsurge in new cases in week 48 of 2021 (first week of December), with 2,940 new cases, an increase of 47% compared with the last week of November 2021. This upward trend in new cases was intensified and became constant from the fourth week of December 2021, with 25,032 new cases. In the second and third weeks, new cases decreased by 39% and 71%, respectively, compared with the first week. This decline in new cases was constant through March 31, 2022. Between December 2021 and March 2022, the period covering the fourth epidemic wave in west Africa, a total of 184,876 new cases and 1,180 deaths were reported. Compared with the previous wave, this fourth wave was less important and less deadly, with a decrease in new cases (−14%) as well as deaths (−70%). This downward trend in the number of new cases was observed in most west African countries, except in Burkina Faso, Cape Verde, Ivory Coast, and Mali, which experienced a more significant fourth wave with increases of 89%, 121%, 38%, and 312%, respectively. A significant decline in CFRs was noted throughout the west African region (Table 3). After this fourth wave, Nigeria was the country with the highest number of confirmed cases (255,414 confirmed cases), representing 28.3% of the total number of reported cases of COVID-19 in west Africa. It should be highlighted, however, that Nigeria is the most populated African country, with a population of 218.5 million. Together with Ghana, Senegal, and Ivory Coast, it accounted for nearly 65% of COVID-19 cases in the west African region. The remaining 12 countries each reported between 7,000 and 58,000 cases after these four waves. The highest CFR after these four epidemic waves was reported by Liberia, with a CFR of 4.0%, higher than the continental CFR of 2.2%, whereas the lowest CFR (0.6%) was observed in Benin (Table 2).

For the fourth epidemic wave, 3,395 complete genome sequences were produced by west African countries (Supplemental Table 5) except Guinea Bissau, Mauritania, Sierra Leone, and Togo. From the analysis of these sequences, it was demonstrated, according to the Pangolin nomenclature, that 80 lineages were present in west Africa during this period. Among these, 57 were new lineages (Supplemental Table 6) compared with those detected from the complete and high coverage sequences produced during the first three epidemic waves. Different sub-lineages deriving from lineage B.1.1.529 (Omicron) circulated mainly during this wave. The Omicron variant was the main VOC that fueled this epidemic wave in west Africa (Supplemental Figure 2). Lineage BA.1.1 represented 16% of available sequences, followed by lineages BA.1 (15%), BA.1.15 (12%), BA.1.1.11 (7%), and BA.1.1.1 (7%) (Supplemental Figure 1). The greatest number of Omicron lineage sequences was recorded in December 2021 (Figure 3). The presence of the various Omicron lineages was also noted across different west African countries (Supplemental Table 4).

### Mutational analysis of SARS-CoV-2 in west Africa.

Studies carried out in west Africa within the framework of the genomic surveillance of SARS-CoV-2 have made it possible to highlight SARS-CoV-2 genomic variations through different types of mutations.

Nigeria was the first country in west Africa to provide a complete SARS-CoV-2 genome sequence.^38^ Preliminary Nigerian reports were initially focused on the analysis of Spike protein mutations without a full molecular characterization. At the beginning of the pandemic, Happi et al.^39^ described four D614G spike mutations in Nigerian SARS-CoV-2 genomes. Subsequently, a study of 378 complete genomes described the presence of the nonsynonymous mutations N501Y, E484K, Q52R, A67V, D614G, Q677H, F888L, L452R, P681R, and V1104L in the Spike protein as well as two in-frame deletions at positions 69-70 and 144.^34^ An analysis of the distribution of single nucleotide polymorphisms (SNPs) demonstrated that the Nigerian SARS-CoV-2 exhibited 99.9% genomic similarity with four major conserved genomic regions and contained 66 SNPs, of which 31 were informative.^40^ An average frequency of 2.22 SNPs per 1,000 nucleotides (nts) was found in Nigerian SARS-CoV-2 genomes. ORF10 was the region of the genome with the highest density of SNPs (8.55 SNPs/1,000 nts). In the *S* gene, the D614G mutation was found to be present in 61.1% of the genomic sequences analyzed.^40^

In Ghana, during the first epidemic wave, a comprehensive molecular epidemiological analysis of SARS-CoV-2 genomic data identified a lower-than-expected mutation rate (2.5 nucleotides per month). However, a significant number of synonymous and nonsynonymous mutations were found: The D614G spike variant and mutations in the nucleocapsid were found in 55% and more than 70% of the genomes. ORF14 contained variants in more than 75% of the genomes, and ORF1a had nonsynonymous mutations in nearly 50% of the genomes. Nonsynonymous mutations had the highest diversity of any gene/ORF. New local mutations were also found in this study. The Spike mutation D614G that appeared to be correlated with high transmissivity and mortality at that time was associated with lower levels of transmission during the first epidemic wave in Ghana.^41^ During the evolution of the disease in Ghana, the genetic diversity of the virus has been high. A mutational analysis of amino acid substitutions on 1,077 positive samples showed the circulation of more than seven lineages with an average of 30 mutations each. The Spike D614G mutation was the most abundant, representing 98% of the mutations, followed by ORF1b P314L (92%). Three highest individual mutations (Q52R, Q677H, and F888L) were encountered in the Eta variant spike protein. Among the mutations shared between the different lineages, the fitness substitutions D614G and P681R/H had the highest frequency.^42^

In Guinea Conakry, this genetic diversity of SARS-CoV-2 has also been demonstrated using strains from different locations and obtained at different times. Nucleotide sequence analysis has found strong genetic heterogeneity between strains circulating at the same time. The D614G mutation was present in all sequences. Samples assigned to the Alpha genotype, in addition to markers specific of this variant, contained additional substitutions: E619K, P9S, Q965H, S12C, T76N, Q965H, and D138Y. A common set of mutations unique to the *Spike* gene (K182R, L452R, T478K, D614G, P681H, D796Y) was also documented.^43^

In Senegal, a study evaluating the genetic evolution of SARS-CoV-2 between the first and second waves detected new combinations of Spike mutations, including E484K + N501T, L452R + N501Y, and L452M + S477N, that were exclusively present during the second wave.^31^

In Mali, the first two sequenced and analyzed genomes detected 33 mutations, of which 20 were nonsynonymous, 11 were synonymous, and 2 were in the 5′-untranslated region of the first sample. At the level of the spike protein, the D614G mutation was found for one sample and the D614N mutation for the second.^44^ Subsequent sequence analysis also identified minor synonymous and nonsynonymous variations in the genome of the virus circulating in Mali. These SNPs, associated with the Wuhan reference strain and strains that have evolved from it, were previously identified around the world, suggesting different and independent introductions of the virus into Mali. Further characterization of the genomics of the Malian strains demonstrated a 3–amino acid microdeletion of the ORF1a protein, previously unidentified in Africa at the beginning of the pandemic. This characterization also allowed the detection of quasi-species indicated by the presence of at least two isolates at positions 14,408 and 18,973 of two Malian strains.^29^

In Benin, after 1 year of circulation of the virus in the country, an analysis of the SARS-CoV-2 genomic diversity was performed.^33^ A pre-selection of mutations identified as having effects on the viral phenotype was first performed using SNP tests based on reverse transcription-polymerase chain reaction (PCR). This study detected characteristic Spike protein mutations: the 69/70 deletion and the E484K, N501Y, P681H, L452R, K417N, or P681R mutation. Complete genome sequencing revealed the presence of 229 nonsynonymous nucleotide substitutions across the genome with 57 (24.9%) of these mutations present in the Spike protein.^33^ During the emergence of the “Indian” variant in the country, two sub-lineages of this variant were detected: B.1.617.2 and AY.4. In the genomes of the Beninese Delta variants, none of the signature mutations V70F, W258L, and K417N or the E484K mutation associated with immune evasion were detected. In addition, the frequency of occurrence of mutations in Delta variant genomes varied between circulating strains.^37^

In Niger, a study conducted early in the pandemic reported the genetic evolution of SARS-CoV-2 in the country from confirmed COVID-19 cases with mutations characteristic of the first clade that emerged from Wuhan.^45^ Mutations present in other SARS-CoV-2 genomes that emerged from various parts of the world were also present in Niger strains. However, a mutation specific to Niger genomes (C18959T) was discovered in this study. Thus, a change of amino acid alanine (A) to valine (V) in ORF1ab was caused by this mutation. In addition, the silent mutation A361G, present in neighboring Niger, was common to the cluster in this study. This suggested that these genomes in Niger were part of a cluster of recent African ancestry.^45^

In the Gambia, mutational analysis of reinfection cases revealed more mutations in the *Spike* gene during reinfection than during the first infection. The nonsynonymous mutations reported in the reinfection cases were L18F, N440K, D614G, G946V, L452M, P681M, and A1020S.^46^

These different data on circulating strains of SARS-CoV-2 in west Africa reflect their high genetic variability. Overall, a similarity was observed between the different mutations documented. The D614G mutation in the Spike protein was the most common mutation.

## DISCUSSION

The west African region is the third most affected region on the continent after South Africa and East Africa, accounting for 8% of cumulative confirmed cases on the continent and 0.2% globally.^47^ The cumulative number of infections as well as the mortality rate were relatively low in west Africa.^51^ COVID-19 has affected the various countries in the west African region in different ways depending on the realities of the countries and the implemented response measures. Despite alarming predictions of the spread of SARS-CoV-2 in Africa,^52^ the evolution of the pandemic has been very slow, unlike in Europe and the Americas. Several hypotheses have been put forward to explain this slow spread, probably due to various factors, including genetic and environmental factors, experience with pandemics, low diagnostic capacity, and the youth of the population.^53^^,^^54^ Six months into the pandemic in Africa, only 12 countries in the WHO African region had reached the desirable threshold of 10 tests per 10,000 people per week.^55^ Seroprevalence rates found in studies conducted in west Africa suggest that reported cases clearly underestimated the true burden of disease. It is therefore wise to analyze these epidemiological data with caution. A recent study conducted in Africa demonstrated that mortality in critically ill patients with COVID-19 was higher in Africa than in other continents.^56^

Mutational analysis of SARS-CoV-2 documented through studies carried out in west Africa has revealed considerable genetic heterogeneity. Synonymous and nonsynonymous mutations were the most frequent genetic variations. A study that sought to determine the mutations and behavioral pattern of SARS-CoV-2 in Africa also demonstrated the predominance of nonsynonymous mutations.^57^ Through these studies, it was also shown that mutations occurred in various regions spanning the genome. However, the D614G mutation in the Spike protein detected between January and February 2020 in China and Europe^58^ was the most common mutation described in studies conducted in west Africa. This D614G mutation, associated with high infectivity and higher viral shedding, was not only common in west Africa. It was the characteristic mutation of all variants that emerged from the Wuhan reference strain.^59^^,^^60^ It was also the common peak protein mutation observed on all continents, with a large-scale analysis demonstrating a prevalence of 99.1%.^61^^,^^62^ The D614G mutation was considered the most widespread mutation in whole genomes sequenced worldwide and the most frequently established in Europe, Asia, the Americas, and Australia.^63^^,^^64^

In other parts of Africa, the predominance of the D614G mutation was comparable to that found in west Africa. In South Africa, studies carried out during the first wave of infection reported a predominance of the D614G mutation.^65^ It was found in 99% of South African sequences when new lineages were identified during the first wave.^66^ In north Africa, the D614G mutation dominated sequences at the start of the pandemic, with a prevalence of 78.7%.^67^ In studies carried out in Central Africa, high observance of the D614G mutation was reported in genomes sequenced during the first wave.^68^^–^^70^ In east Africa, the D614G mutation also dominated the onset of the pandemic.^71^^–^^74^ This similarity in mutational pattern between countries reflects the circulation of viral strains across borders. Mutational analysis of west African strains of SARS-CoV-2 has enabled us to highlight the wide variation in mutation numbers between populations, which has strongly influenced the emergence of different SARS-CoV-2 lineages in west Africa.

Regarding the genetic profile of SARS-CoV-2 in west Africa, the few studies carried out and the analysis of the sequences deposited in GISAID have demonstrated the presence of great diversity. Although not all sequences available in GISAID were included in the analysis because of sequence selection based on genome completeness and high sequencing coverage, the data used in this study allowed us to estimate representative prevalence of the various SARS-CoV-2 lineages in west Africa.

The analysis of the evolutionary diversity based on metadata analysis allowed us to enumerate 203 circulating lineages in west Africa from 8,650 sequences produced between February 2020 and March 2022, according to the Pangolin nomenclature. Despite the small number of genomic sequences produced compared with the numbers of confirmed COVID-19 cases reported in west Africa, a real genetic diversity of the virus could be observed. A similarity was observed between the lineages circulating in each epidemic wave across countries, suggesting a regional spread of the virus. The strains circulating in west Africa were also present in other parts of the continent and in the rest of the world.^75^

During the early phase of the pandemic, there was a predominance of lineages A and B.1 in west Africa. This same profile was found in a phylogenetic and phylogeographic analysis conducted in 33 African countries, including 11 west African countries.^76^ These various SARS-CoV-2 clades that circulated in west African countries originated from China and Europe.^48^ As the pandemic evolved, genomic surveillance was intensified in west Africa and played a major role in the detection of lineages classified as VOCs by the WHO. A study of global variant transmission showed that as of June 2021, Africa accounted for 2% of countries with identified SARS-Cov-2 variants. At this date, African countries reporting circulation of the Alpha variant accounted for 20%, 27% for the Beta variant, 26% for the Eta variant, 11% for the Delta variant, and no cases for the Gamma variant.^50^ The lineages of concern were initially detected outside west Africa. Their presence and spread in the west African region suggest successive viral introductions as well as, in at least one case, exportations. Indeed, Wilkinson et al.^76^ demonstrated the emergence and spread in Africa of several VOIs and VOCs, 64% of which originated from Europe. Except for the first epidemic wave, each phase of the disease was characterized by a dominant VOC. The VOC Alpha was predominant during the second epidemic wave (November 2020–May 2021), VOC Delta during the third epidemic wave (June 2021–November 2021), and VOC Omicron in the fourth epidemic (December 2021–March 2022). These VOCs were reported in most cases during phases of significant disease progression. In most countries, they were associated with high disease transmissibility, leading to a sharp rise in the number of cases. This consequence of VOCs is combined with their main characteristics, which are increased transmissibility or virulence, reduced neutralization by antibodies obtained through natural infection or vaccination, and the ability to escape detection or reduced therapeutic or vaccine effictiveness.^77^ Unlike other VOCs, the circulation of VOC Omicron in west Africa has been associated with a decrease in confirmed cases and deaths. This could be related to the highly mutated nature of Omicron, which reduced its virulence.^78^^,^^79^ In other parts of the world, the downward trend in cases and deaths associated with Omicron has also been observed. Serious outcomes from COVID-19 have been reduced mainly because of the protection conferred by previous infection and/or vaccination.^80^^,^^81^

In other continents such as Asia, Europe, Oceania, and the Americas, a similar period of VOC circulation to that observed in west Africa has been reported through an analysis conducted on SARS-CoV-2 variants.^82^

In North Africa, VOCs began to appear in February 2021. In most North African countries, VOC Alpha was the predominant variant between March and April 2021. The VOC Delta was the majority line between June and July 2021.^83^^,^^84^ In southern African countries, the period of VOC circulation was similar to that found in west Africa. A difference was noted in the pandemic profile of the second wave, which was dominated by VOC Beta. The third and fourth epidemic waves were dominated by VOCs Delta and Omicron, respectively.^85^ In east African countries, VOCs Alpha, Beta, Delta, and Omicron circulated there with a considerable contribution compared with the occurrence of the different epidemic waves. The circulation period of VOCs Alpha, Delta, and Omicron was similar to that of west Africa.^86^^–^^88^ The same VOC circulation pattern has been found in central Africa.^68^^,^^69^^,^^89^

This similarity in the period of VOC circulation between west African countries and other parts of the world reflects an introduction of viral strains. At the start of the pandemic in west Africa, the first emerging variants of the Wuhan reference strain, predominant in Senegal, the Gambia, Nigeria, and Ghana, originated from Europe. Similarly, VOC Alpha was introduced to these countries from Europe.^75^ The introduction of Delta VOC was mainly attributed to India, mainland Europe, the United Kingdom, and the United States.^90^ In addition to these viral introductions, there have been reports of the spread of SARS CoV-2 variants from sub-Saharan Africa. During the first epidemic wave, it was reported that strains that had circulated in Senegal and the Gambia evolved genetically there before reaching the Maghreb and spreading to Marseille. This variant, called Marseille 1, caused a brief epidemic in Marseille in July 2020.^91^ A phylogeographic reconstruction enriched with African sequences demonstrated that more than 85% of intercontinental exchanges of VOC Alpha in Africa originated in west African countries. For the VOCs Delta and Omicron, phylogeographic reconstructions centered on Africa have demonstrated that routes of dissemination of these variants involved all regions of the continent spatially.^90^

## CONCLUSION

In this work, we reviewed the epidemiological data of SARS-CoV-2 infection and the genetic diversity of the virus in the 16 west African countries. First, it was noted that the pandemic evolved slowly in these countries, as in most other African countries. The pandemic developed successively in four phases. In each west African country, specific disease incidences and CFRs were observed. A review of the west African literature on sociodemographic factors showed that gender and age were influential factors in the occurrence and mortality of COVID-19 in west Africa. Second, mutational analysis of circulating strains showed that mutations common to different regions of the world were also distributed in west Africa, thus demonstrating the adaptability of SARS-CoV-2 in this region. Finally, the review of SARS-CoV-2 genome sequence studies conducted in west Africa as well as the analysis of sequence data produced and deposited in GISAID allowed us to identify distinct viral lineages until the end of March 2022. Despite this important repertoire of the genetic diversity of SARS-CoV-2, most studies were found to be descriptive of the mutations present, but not in-depth analytical of the real impact and understanding of these mutations on the transmissibility and pathogenesis of SARS-CoV-2 in west Africa.

## Supplemental Materials


Supplemental materials
