# Supplementary material for: COVID-19 in 16 West African Countries: An Assessment of the Epidemiology and Genetic Diversity of SARS-CoV-2 after Four Epidemic Waves
Source: Am J Trop Med Hyg. 2023 Aug 28;109(4):861–73. doi: 10.4269/ajtmh.22-0469 (PMC10551082; doi:10.4269/ajtmh.22-0469)

Table S1: Geographical distribution of the top 15 SARS CoV-2 lineages of the first wave in West Africa

Lineages	Benin	Burkina Faso	Ivory Coast	Gambia	Ghana	Guinea	Guinea-Bissau	Mali	Niger	Nigeria	Senegal	Sierra Leone	Togo
A													
A.19													
A.21													
B.1													
B.1.1													
B.1.1.1													
B.1.1.242													
B.1.1.359													
B.1.1.462													
B.1.1.466													
B.1.1.485													
B.1.1.487													
B.1.292													
B.1.336													
B.1.416													

*Blue box = presence
*White box = absence

Table S2: Geographical distribution of the top 15 SARS CoV-2 lineages of the second wave in West Africa

Lineages	Benin	Burkina Faso	Cabo Verde	Ivory Coast	Gambia	Ghana	Guinea	Guinea-Bissau	Liberia	Mali	Mauritania	Niger	Nigeria	Senegal	Sierra Leone	Togo
A.21																
A.27																
B																
B.1																
B.1.1																
B.1.1.420																
B.1.1.487																
B.1.1.7																
B.1.351																
B.1.416																
B.1.525																
B.1.617.2																
L.3																
R.1																
B.1.1.404																

*Blue box = presence
*White box = absence

Table S3: Geographical distribution of the top 15 SARS CoV-2 lineages of the third wave in West Africa

Lineages	Benin	Cabo Verde	Ivory Coast	Gambia	Ghana	Guinea	Liberia	Mali	Mauritania	Niger	Nigeria	Senegal	Sierra Leone	Togo
AY.109	■				■	■					■			
AY.122	■				■						■	■		■
AY.34	■	■	■			■		■			■	■		■
AY.34.1		■						■	■			■		
AY.36	■		■		■	■					■			■
AY.37	■	■			■	■	■			■	■			■
AY.39	■				■						■			
AY.4					■						■		■	
AY.70	■									■	■			
AY.88					■						■			■
AY.99											■			
B.1.1.7	■		■	■	■	■			■		■			
B.1.525			■		■	■					■	■		■
B.1.617.2	■		■	■	■	■					■	■	■	■
B.1		■	■								■	■		■

*Blue box = presence
*White box = absence

Table S4: Geographical distribution of the top 15 SARS CoV-2 lineages of the fourth wave in West Africa

Lineages	Benin	Burkina Faso	Cabo Verde	Ivory Coast	Gambia	Ghana	Guinea	Liberia	Mali	Niger	Nigeria	Senegal
BA.1.1.15												
BA.1.18												
BA.1.13												
BA.1.15.1												
BA.1.17												
BA.1.1.14												
AY.36												
BA.1.1.9												
BA.1.17.2												
BA.1.14												
BA.1.1.1												
BA.1.1.11												
BA.1.15												
BA.1												
BA.1.1												

*Blue box = presence
*White box = absence

Table S5: Number of complete, high coverage sequences produced by the 16 West African countries.

	1st wave	2nd wave	3rd wave	4th wave *
	**Sequences generated (n)**	**Sequences generated (n)**	**Sequences generated (n)**	**Sequences generated (n)**
**Benin**	12	221	74	80
**Burkina Faso**	62	110	-	17
**Cabo Verde**	-	20	33	164
**Ivory Coast**	44	265	68	65
**Gambia**	233	19	11	168
**Ghana**	82	405	297	583
**Guinea**	43	45	54	189
**Guinea-Bissau**	2	3	-	-
**Liberia**	-	14	43	34
**Mali**	25	11	2	1
**Mauritania**	-	5	10	-
**Niger**	15	36	2	54
**Nigeria**	192	396	1396	1840
**Senegal**	267	237	224	200
**Sierra Leone**	10	22	2	-
**Togo**	29	92	122	-
**Total**	**1016**	**1901**	**2338**	**3395**

Source : epicov.org/epi3/frontend

*High coverage [<1% Ns (undefined bases)] not included in Wave 4

Table S6: Different SARS-COV-2 lineages circulating in West Africa between February 2020 and March 2022

Lineages 1st wave		Lineages 2nd wave		Lineages 3rd wave		Lineages 4th wave		
A	B.1.1.200	A	B.1.351	A.18	AY.5.4	A	B.1.638	BA.1.4
A.18	B.1.1.242	A.18	B.1.525	A.21	AY.70	AY.100		BA.1.6
A.19	B.1.1.254	A.19	B.1.617.2	A.27	AY.91	AY.109	BA.1	BA.1.7
A.21	B.1.1.411	A.21	R.1	AY.100	B.1.640.1	AY.112	BA.1.1	BA.1.9
AY.34.1	B.1.1.462	AY.34.1	A.23.1	AY.122	AY.1	AY.121	BA.1.1.1	BA.2
AY.36	B.1.1.466	AY.36	AY.59	AY.34	AY.103	AY.122	BA.1.1.10	BA.2.10
B	B.1.1.484	B	B.1.1.220	AY.34.1	AY.107		BA.1.1.11	BA.2.12
B.1	B.1.1.71	B.1	B.1.1.28	AY.36	AY.108	AY.133	BA.1.1.12	BA.2.16
B.1.1	B.1.1.8	B.1.1	B.1.1.306	AY.37	AY.111	AY.20	BA.1.1.13	BA.2.3
B.1.1.1	B.1.22	B.1.1.1	B.1.1.519	AY.4	AY.114	AY.34.1	BA.1.1.14	BA.2.5
B.1.1.10	B.1.225	B.1.1.10	B.1.1.59	AY.65	AY.124	AY.36	BA.1.1.15	BA.2.6
B.1.1.359	B.1.292	B.1.1.359	B.1.1.83	AY.88	AY.125	AY.36.1	BA.1.1.16	BA.2.7
B.1.1.404	B.1.298	B.1.1.404	B.1.177.81	B	AY.126	AY.39	BA.1.1.17	BA.2.9
B.1.1.409	B.1.336	B.1.1.409	B.1.177.86	B.1	AY.128	AY.4	BA.1.1.2	BA.3
B.1.1.420	B.1.356	B.1.1.420	B.1.214.2	B.1.1	AY.16	AY.43	BA.1.1.3	C.37.1
B.1.1.485	B.1.379	B.1.1.485	B.1.351.2	B.1.1.318	AY.27	AY.5.4	BA.1.1.5	
B.1.1.487	B.1.388	B.1.1.487	B.1.351.5	B.1.1.404	AY.3	AY.70	BA.1.1.6	
B.1.1.7	B.1.398	B.1.1.7	B.1.36	B.1.1.7	AY.33	AY.88	BA.1.1.7	
B.1.160	B.1.428.2	B.1.160	B.1.36.29	B.1.351	AY.38	AY.91	BA.1.1.8	
B.1.177	B.1.438	B.1.177	B.1.361	B.1.525	AY.4.2	B	BA.1.1.9	
B.1.2	B.1.441	B.1.2	B.1.369	B.1.617.2	AY.44	B.1	BA.1.10	
B.1.243	B.1.466	B.1.243	B.1.466.1	L.3	AY.45	B.1.1	BA.1.13	
B.1.416	B.1.467	B.1.416	B.1.509	R.1	AY.46	B.1.525	BA.1.13.1	
B.1.462	B.1.476	B.1.462	B.1.526	AY.109	AY.5	B.1.617.2	BA.1.14	
L.3	B.1.482	L.3	B.1.544	AY.112	AY.55		BA.1.15	
A.11	B.1.582	A.27	B.1.597	AY.121	AY.6	AY.113	BA.1.15.1	
A.12	B.1.8	AY.100	B.1.617.1		AY.75	AY.30	BA.1.16	
A.2	B.1.9	AY.122	B.1.629	AY.133	AY.98	AY.4.2.3	BA.1.17	
A.3	B.28	AY.34	C.16	AY.20	AY.98.1	AY.47	BA.1.17.2	
AE.1	B.3	AY.37	C.36	AY.36.1	AY.99	AY.87	BA.1.18	
B.1.1.117	B.40	AY.4	C.36.3	AY.39	B.1.351.3	AY.9.2	BA.1.19	
B.1.1.174	B.6	AY.65	L.2	AY.43	B.1.620	B.1.1.161	BA.1.20	
		AY.88	Q.4			B.1.1.263	BA.1.21	
		B.1.1.318				B.1.242	BA.1.21.1	

Figure S1: Top 15 major lineages of SARS-CoV-2 in each epidemic wave in West Africa

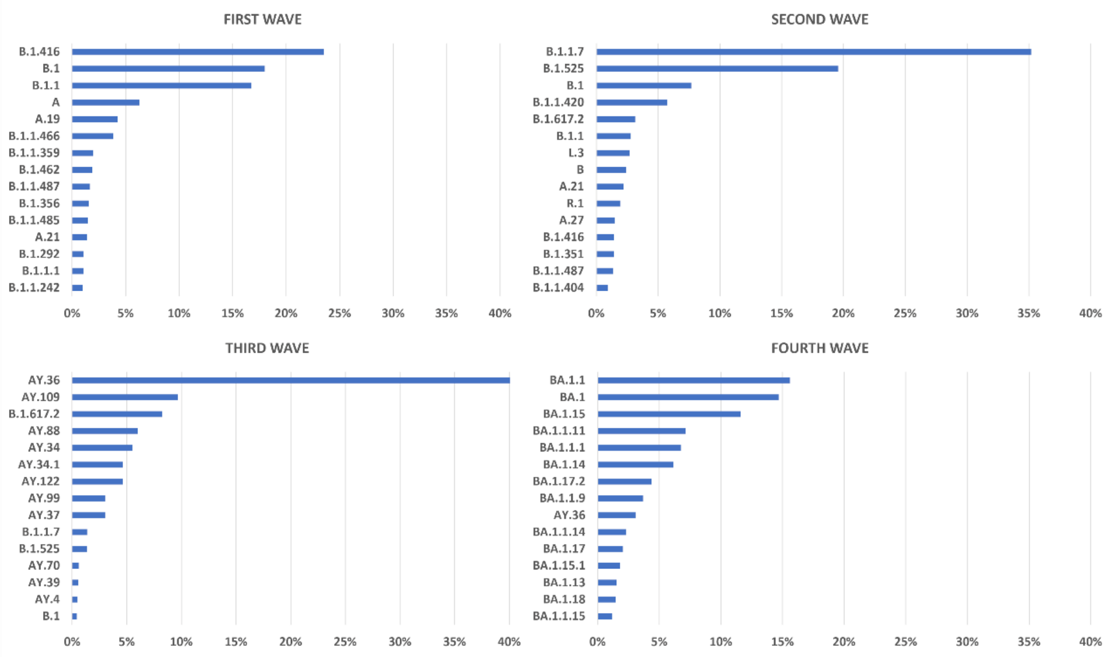

Figure S2: Distribution of VOCs and non-VOCs in West Africa on March 31, 2022.

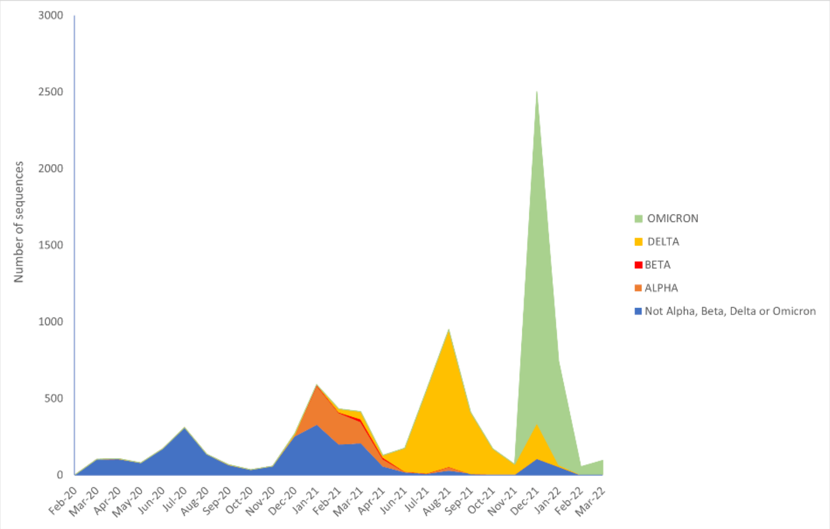

Supplement: Supplementary file 1 [file tpmd220469.SD1.pdf]
